# Mobility measures and waking-day movement behaviour composition among older adults: a compositional data analysis of the McMaster Monitoring My Mobility study

**DOI:** 10.1186/s12966-026-01939-4

**Published:** 2026-06-23

**Authors:** Shawn Hakimi, Renata Kirkwood, Stuart M. Phillips, Rong Zheng, Marla K. Beauchamp

**Affiliations:** 1 School of Rehabilitation Science, Faculty of Health Science, Institute for Applied Health Sciences, McMaster University, 1400 Main Street West, Hamilton, Ontario L8S 1C7 Canada; 2 Department of Kinesiology, Faculty of Science, McMaster University, 1280 Main Street West, Hamilton, Ontario L8S 4K1 Canada; 3 Department of Computing and Software, Faculty of Engineering, McMaster University, 1280 Main Street West, Hamilton, Ontario L8S 4L7 Canada

**Keywords:** Physical activity, Sedentary behaviour, Time-use epidemiology, Ageing, Physical functioning, Quality of life, Locomotor, Ambulation, Geriatric

## Abstract

**Background:**

Mobility is a cornerstone of healthy ageing typically assessed using self-report and performance-based measures. Recent research suggests these measures are poor proxies for real-world mobility. Device-derived measures of real-world mobility reflect the relative proportions of time a person allocates to sedentary behaviour (SB), light-intensity physical activity (LIPA), and moderate-to-vigorous physical activity (MVPA) that together form the waking-day movement behaviour composition. The aim of this study was to examine the associations between commonly used mobility measures and the waking-day movement behaviour composition in older adults.

**Methods:**

A cross-sectional analysis of data from over 1200 older adults ≥ 65 years of age from the baseline cohort (2022–2024) of the McMaster Monitoring My Mobility (MacM3) study was conducted. Compositional data analysis was used to examine the associations between 6 self-report and 10 performance-based measures of mobility and multiple components of the waking-day movement behaviour composition and establish differences in movement behaviours according to different levels of mobility. Examples of included measures are the Physical Activity Scale for the Elderly (PASE) and Timed Up and Go (TUG).

**Results:**

Data from 1227 older adults (73.6 ± 5.3 years) were analysed. Significant differences in waking-day movement behaviour composition were found across the self-report and performance-based mobility measures. Older adults with lower mobility accumulated more SB and less MVPA throughout the day. For example, those with poorer TUG performance accumulated 34 more minutes/day of SB, and 22 min/day less of MVPA compared to those with better performance. The PASE, 400-meter walk, and fast gait speed tests were the strongest indicators of real-world mobility; however, the effect sizes were small. No measure showed a significant association with LIPA.

**Conclusions:**

Traditional self-report and performance-based measures of mobility are associated with the waking-day movement behaviour composition in older adults; however, their ability to explain real-world mobility is limited and varies across measures. The lack of association between any of the measures and LIPA is surprising, given that older adults spend most of their time in this activity intensity. These findings challenge the widespread use of traditional mobility measures to make inferences about real-world mobility.

**Supplementary Information:**

The online version contains supplementary material available at https://doi.org/10.1186/s12966-026-01939-4.

## Background

Mobility is a cornerstone of healthy ageing regarded by ageing experts as the ‘6th vital sign’ [1]. Mobility limitations such as difficulty walking, bending, or climbing several flights of stairs are a strong predictor of falls, hospitalisation, institutionalisation and death in older people [2]. Although mobility has been defined in various ways [3], the World Health Organization (WHO) more recently defines it as “movement in all its forms whether powered by the body (with or without an assistive device) or a vehicle” [4], building on its earlier definition within the International Classification of Functioning, Disability and Health (ICF) framework as “changing body position or location or by transferring from one place to another, by carrying, moving or manipulating objects, by walking, running, or climbing, and by using various forms of transportation” [5]. Together, these definitions reflect a multidimensional construct, which underscores the need for comprehensive approaches for measuring mobility in older adults [6].

In practice, mobility is often assessed using a range of self-report and performance-based measures [6–8]. Self-report measures typically capture perceived ability (e.g., Late Life Function and Disability Instrument [LLFDI]) and/or frequency and extent of movement across environments (e.g., Life Space Assessment [LSA]), while performance-based measures assess physical capacity for mobility (e.g., Timed Up and Go [TUG]). Although these measures reflect different dimensions of mobility, they are frequently used (sometimes interchangeably) as proxies for overall mobility in clinical and research settings. However, the extent to which these measures reflect real-world, everyday mobility remains unclear [9, 10]. Several studies suggest they may be poor proxies, as they are prone to measurement error, conducted under standardised conditions, initiated by external cues, and may not reflect purposeful, real-world behaviour [11–13].

Real-world mobility, typically measured using wearable devices, reflects the observable expression of mobility in daily life, that is, what an individual actually does in free-living conditions. Within a multidimensional framework, this represents the enacted domain of mobility and is shaped by interactions between physiological, intrinsic, and environmental factors [14, 15]. It is operationalised as the distribution of time across movement behaviours: sedentary behaviour (SB), light-intensity physical activity (LIPA), and moderate-to-vigorous physical activity (MVPA) which together comprise the waking-day movement behaviour composition. Accordingly, it remains unclear whether commonly used self-report and performance-based measures of mobility, which primarily capture perceived ability or physical capacity, adequately reflect this real-world expression of mobility.

Historically, research on movement behaviours in older adults has focussed on physical activity, namely LIPA and MVPA [16, 17]; however, the time older adults spend in these behaviours only account for small portions of the day, 18% and 3% respectively [18–21]. Researchers now appreciate the relevance of the movement intensities that make up the remainder of the day including SB (e.g., screen time) [22]. Recent research demonstrates that the entire daily movement behaviour composition has implications for health [22, 23]. Several studies have noted that physical activity and SB can be impacted by physical functioning, gait, and balance [24–29]. While studies have examined associations between mobility measures and physical activity and SB on their own, research has not yet addressed the extent of these associations in relation to all components of the daily movement behaviour composition.

Statistical approaches that assess the association between mobility and movement behaviours should account for their compositional properties [30, 31]. Traditional statistical approaches analyse movement behaviours independently which is problematic and may lead to distorted results because movement behaviours are co-dependent whereby their summed time always equates to a fixed amount of time (e.g., waking-day hours) [30–33]. That is, if time in one movement behaviour changes, an equal and opposite change must occur in another or combination of remaining movement behaviours [30–33]. Compositional data analysis (CoDA) statistical techniques are appropriate for analysing data that is a composition of a finite whole, such as movement behaviours in the waking-day [33]. 

An integrated approach to understanding the associations between mobility measures and movement behaviour has implications for informing healthy ageing initiatives and identifying modifiable aspects of mobility that beneficially influence how individuals distribute time across daily movement behaviours. The objectives of this study are to use a CoDA framework to: (1) examine the extent to which commonly used self-report and performance-based measures of mobility are associated with multiple components of the waking-day movement behaviour composition and, (2) understand how older adults with different levels of mobility allocate their time in waking-day movement behaviours.

## Methods

### Study design and participants

This study is a cross-sectional analysis of baseline data from the McMaster Monitoring My Mobility (MacM3) study, an ongoing prospective cohort study tracking later life mobility trajectories using wearable technology [34]. The study includes 1,506 older adults from Southern Ontario, Canada. Baseline data were collected from May 2022 to June 2024. Inclusion criteria were ≥ 65 years of age, community-dwelling, provision of informed consent, absence of visual or hearing impairments that would make participation unsafe, ability to speak and understand English, and being free from major mobility limitation at baseline as determined by the Preclinical Mobility Limitation scale (PCML) [35]. Ethics approval was obtained from the Hamilton Integrated Research Ethics Board at McMaster University. Participants were largely recruited from the community through word of mouth, newspapers, and Facebook ads; a small contingent came from a pre-recruited sample from a larger intergenerational study on ageing [36].

### Mobility measures

The explanatory variables are measures of mobility obtained by trained research staff: (i) self-report measures—via standardised questionnaires administered through phone-call assessments or in-person interviews; (ii) performance-based measures—a battery of physical tests collected in-person at one of two data collection sites. A detailed description of the MacM3 protocol has been published elsewhere [37].

Table 1 summarises the mobility measures, briefly describes them (refer to the reference provided for detailed information), and lists the data source. To facilitate comparison among ‘low’, ‘moderate’ and ‘high’ levels of mobility, scores for each mobility measure were grouped into tertiles [38]. In addition to examining the individual mobility measures, indices were created for each category of measure. To do that, percentile ranks of each of the individual measures were created and averaged; then, tertiles were created based on these averages. Boundary values for each tertile for all mobility measures and indices are provided in Supplementary Table A1.

**Table 1:** Overview of mobility measures

Name of measure	Explanation of measure and reference for measurement protocol	Exposure groups	Data source
	Self-report measures		
Preclinical Mobility Limitation Scale	Self-reported assessment of three preclinical mobility limitations (walking 0.5 km, walking 2 km, climbing up stairs)^a^	Predefined groups	Questionnaire
Late-Life Function and Disability Instrument-Disability Component	Self-reported frequency of performing 16 life tasks and the person’s limitation in their capacity to perform those tasks^b,c^	Tertiles	Questionnaire
Late-Life Function and Disability Instrument-Physical Function Component	48 items that assess self-reported difficulty in performing discrete activities via three domains: advanced lower extremity functioning, basic lower extremity functioning and upper extremity functioning^b,c^	Tertiles	Questionnaire
Physical Activity Scale for the Elderly	12-item questionnaire that measures amount of physical activity undertaken in a 1-week period^d^	Tertiles	Questionnaire
Life Space Assessment	Life-space mobility (i.e., mobility in the home and community) measured in three sections: life-space level, frequency, independence^e^	Tertiles	Questionnaire
Mobility Assessment Tool-Short Form	Animated video clips showing both the nature and demands of various tasks (e.g., climbing stairs); participant rates their ability to do that task^f^	Tertiles	Computer-based assessment
	Performance-based measures		
Timed Up and Go (usual pace)	Get up from chair, walk 3-meters, turn around, walk back and return to seated chair position at usual walking speed^g^	Tertiles	Physical assessment
Timed Up and Go (fast pace)	Same test described above but at fast walking speed^g^	Tertiles	Physical assessment
Timed Up and Go (cognitive)	Same test as described initially while performing a cognitive task (e.g., counting backwards by two from random number between 50–90)^g^	Tertiles	Physical assessment
Sit to-Stand Test	Stand up 5-times from chair; starting in seated position with back straight, feet on the floor and arms crossed against chest, finished at full-hip extension^h^	Tertiles	Physical assessment
Gait speed (usual pace)	Walking 3-meters at usual pace^i^	Tertiles	Physical assessment
Gait speed (fast pace)	Walking 3-meters as fast as possible^j^	Tertiles	Physical assessment
400-meter walk	Walk 400-meters as fast as possible (terminated if > 15 min)^k^	Tertiles	Physical assessment
Single-Leg Stance	Hands on hips, lift one foot off the floor, and hold the position for as long as possible up to a maximum of 60 s for each leg^l^	Tertiles	Physical assessment
Stair-Climbing Power	Ascend a flight of four stairs with handrails (if needed) as fast as possible^m^	Tertiles	Physical assessment

References—^a^Manty et al. (2007) [35]; ^b^Haley et al. (2002) [39]; ^c^Jette et al. (2002) [40]; ^d^Washburn et al. (1993) [41]; ^e^Baker et al. (2003) [42]; ^f^Rejeski et al. (2015) [43]; ^g^Barry et al. (2014) [44]; ^h^Jones et al. (1999) [45]; ^i^ Abellan Van Kan et al. (2009) [46]; ^j^Bohannon, R.W. (1997) [47]; ^k^Georgiopoulou et al. (2017) [48]; ^l^Springer et al. (2007) [49]; ^m^Bean et al. (2007) [50]

### Movement behaviour variables

The outcome variables were the relative proportions of the waking-day comprised of SB, LIPA, and MVPA measured via a wrist-worn smartwatch. The waking-day was defined as the period from the participant waking up until going to sleep. Participants were instructed to remove the watch and charge it at bedtime, at which point all sensors were inactive until the watch was taken off the charger and worn again the following morning.

Participants received the device at the baseline in-person assessment along with detailed instructions on proper device placement, wear time, and handling by trained research staff and were given a printed flyer containing detailed instructions and troubleshooting guidance. After the assessment period, participants mailed the device back using a prepaid postage box. Participants were asked to wear the device on their non-dominant wrist during waking hours for 10 consecutive days for at least 10 h per day except during swimming, bathing or showering. If they were unable to wear it on their non-dominant wrist, the reason (e.g., physical limitation) was recorded.

The MacM3 study uses a custom data collection application with the TicWatch Pro 3 Ultra GPS (Mobvoi, Nanjing, China) validated for capturing raw accelerometry data in older adults [34]. Raw data was processed using the NiMBaLWear analytic pipeline, a validated open-source data analytic pipeline that quantifies activity intensity from raw free-living data collected over multiple days [51]. Data was captured at 50 Hz and presented in gravitational units that was further processed using the NiMBaLWear Pipeline and the Average Vector Magnitude (AVM) of acceleration using 15-second epochs (and a normal filter) to produce counts [51]. Epochs that did not overlap with detected non-wear time were categorised as SB, LIPA, or MVPA by comparing the AVM to epoch length-independent activity intensity cut-points for older adults ≥ 60 years of age [52]. Specifically, thresholds of 42.5 mg (non-dominant wrist) and 62.5 mg (dominant wrist) were used for light activity, while 98.0 mg (non-dominant) and 92.5 mg (dominant) defined moderate activity. These cut-points have been applied in other ageing cohorts and support comparability across studies examining accelerometry-derived activity patterns [52]. Cut points were applied based on the actual wear location (non-dominant wrist or dominant), and passed to NiMBaLWear, which automatically selects the appropriate thresholds. Sleep detection was not performed; therefore, periods of daytime sleep (e.g., napping) may have been classified as SB.

Non-wear time was identified using a modified Van Hees algorithm, based on sustained periods of low acceleration over overlapping 60-minute windows [53]. Windows were advanced in 1-minute increments to improve detection resolution, and additional criteria were applied to account for short, spurious periods of movement within prolonged non-wear intervals, thereby reducing misclassification due to incidental device movement.

Participants with an average wear time of ≥ 10 h with ≥ 4 valid days of accelerometer data were included in the final study sample [54]. Movement behaviour values were calculated as proportions of each participant’s total wear time [33].

### Covariates

Covariates were considered based on their known relationship with mobility measures and one or more movement behaviours. All variables were gathered at baseline through phone call assessment by trained research staff. *Age* (date of birth; years); *sex* at birth (male/female); *body mass index* calculated from weight (kg) and height (cm) (BMI; kg/m^2^); *education level* (no post secondary education, post secondary diploma/degree); *household income level* [low (< *$50*,*000 CDN*), medium (≥ *$50*,*000 but* < *$100*,*000*), high (≥ *$100*,*000*)]; *chronic conditions* (number of doctor diagnosed medical conditions; Supplementary Material Table A2); *cognitive function* [normal/abnormal; Montreal Cognitive Assessment (MoCA) score 0–22 whereby ≥ 19 is normal] [55]; *race* (white or non-white including mixed race); *smoking* (never, former, current); *marital status* (married or common law, single, divorced/separated/widowed). For *alcohol*, responses about daily, weekly and monthly frequency of standard drink consumption were categorised based on Canada’s low risk drinking guidelines [no risk (no drinks), low (≤ 2 drinks per week), moderate (3–6 drinks per week), high (≥ 7 drinks per week)] [56].

### Analysis strategy

*A posteriori* power analysis was conducted indicating that the study sample had 100% power to detect small (0.02), medium (0.15) and large effects sizes (0.35) with α set at 0.05 (Supplementary Figure A1) [57]. Statistical analyses were performed using SPSS v 30.0 (IBM Corp, Armonk, USA).

CoDA was used to determine differences in waking-day movement behaviour composition according to the measures of mobility [58]. Geometric means for the proportion of time spent in each movement behaviour were calculated as they better represent the central tendency of compositional data than arithmetic means. The codependence between movement behaviours can then be assessed using pair-wise log ratio variances between all behaviours [(e.g., variance of ln (SB/MVPA)] and scaled to aid in interpretation$\:\:{\left[\right(e}^{-\:\frac{{t}^{2}}{2}}\left)\right]$ where *t* is any log-ratio variance]. Values for pair-wise log-ratio variances range from zero to one, with those closer to one indicating higher codependence. These values were then represented in a variation matrix.

Subsequently, movement behaviour variables were transformed from their natural space, the constrained simplex S^d^ onto real space where standard statistical techniques can be used. For this step, *isometric log ratios (ilr)* were used to express the waking-day movement behaviour composition as ratios of its parts (i.e., absolute time spent in SB, LIPA, MVPA). Sequential binary partitioning was used to determine the appropriate configuration of *ilr* coordinates for each movement behaviour, so that each one was assessed as the main behaviour in relation to the remaining movement behaviours (e.g., time spent in SB *relative* to LIPA and MVPA) [59, 60]. Waking-day movement behaviour composition allotted into three parts (SB, LIPA, MVPA) was expressed as two *ilr* coordinates that capture the combined distribution of all parts of the composition. For example, *ilr* coordinates for the relative contribution of MVPA were written as:

$$\:{z}_{i1}=\sqrt{\frac{2}{3}}\mathrm{l}\mathrm{n}\left(\frac{{MVPA}_{i}}{\sqrt[2]{{LIPA}_{i}\:x\:{SB}_{i}}}\right)\:{z}_{i2}=\:\sqrt{\frac{1}{2}}\mathrm{l}\mathrm{n}\left(\frac{{LIPA}_{i}}{\sqrt{{SB}_{i}}}\right)$$

When transforming the values into ratios for CoDA, zero values are not tolerated (e.g., no time spent in MVPA). To address that, multiple behaviours are amalgamated into one or imputation of a minimal detectable value is used. However, in this study sample no zero values were present, thereby avoiding the zero issue [61].

Once movement behaviours were transformed, the two *ilr* coordinates of each movement behaviour were entered as the outcome variables in multivariate analysis of variance (MANOVA) models run for each mobility measure. The *p*-value associated with the first coordinate is the one that provides the relevant information of the numerator variable relative to the remaining movement behaviours [33]. The other coordinate variable is used to fit the model. If the MANOVA suggested rejecting the null hypothesis, Hotelling’s *T*-squared test was used to determine which pair of groups were different and *D-1* simultaneous *t*-tests were used to determine which *ilr* coordinate was responsible for the difference [58]. To avoid an artificial increase of Type 1 error, the α level was set to 0.0167 using the Bonferroni correction [58]. The MANOVA results pertaining to the *ilr* coordinates were back-transformed to obtain the amount of time (hours: minutes) for that behaviour relative to the remaining behaviours in the mean waking-day composition. Detailed step-by-step instructions for CoDA analysis are provided elsewhere [62]. Confounding variables were tested against each movement behaviour using univariate linear regression. Those significantly associated with any behaviour based on a conservative *p*-value of < 0.2 were controlled for in the MANOVA models.

Sex and age stratified analyses were run (male/female; mean age split < 73.6 years ≥ 73.6 years) based on their a priori consideration as effect modifiers. The patterns of association were generally the same as the full sample; therefore, final analyses were run on the full sample with sex and age treated as confounding variables. Effect sizes from compositional MANOVA were used to evaluate the strength of association between mobility measures and differences in waking-day movement composition.

## Results

**Fig. 1:**
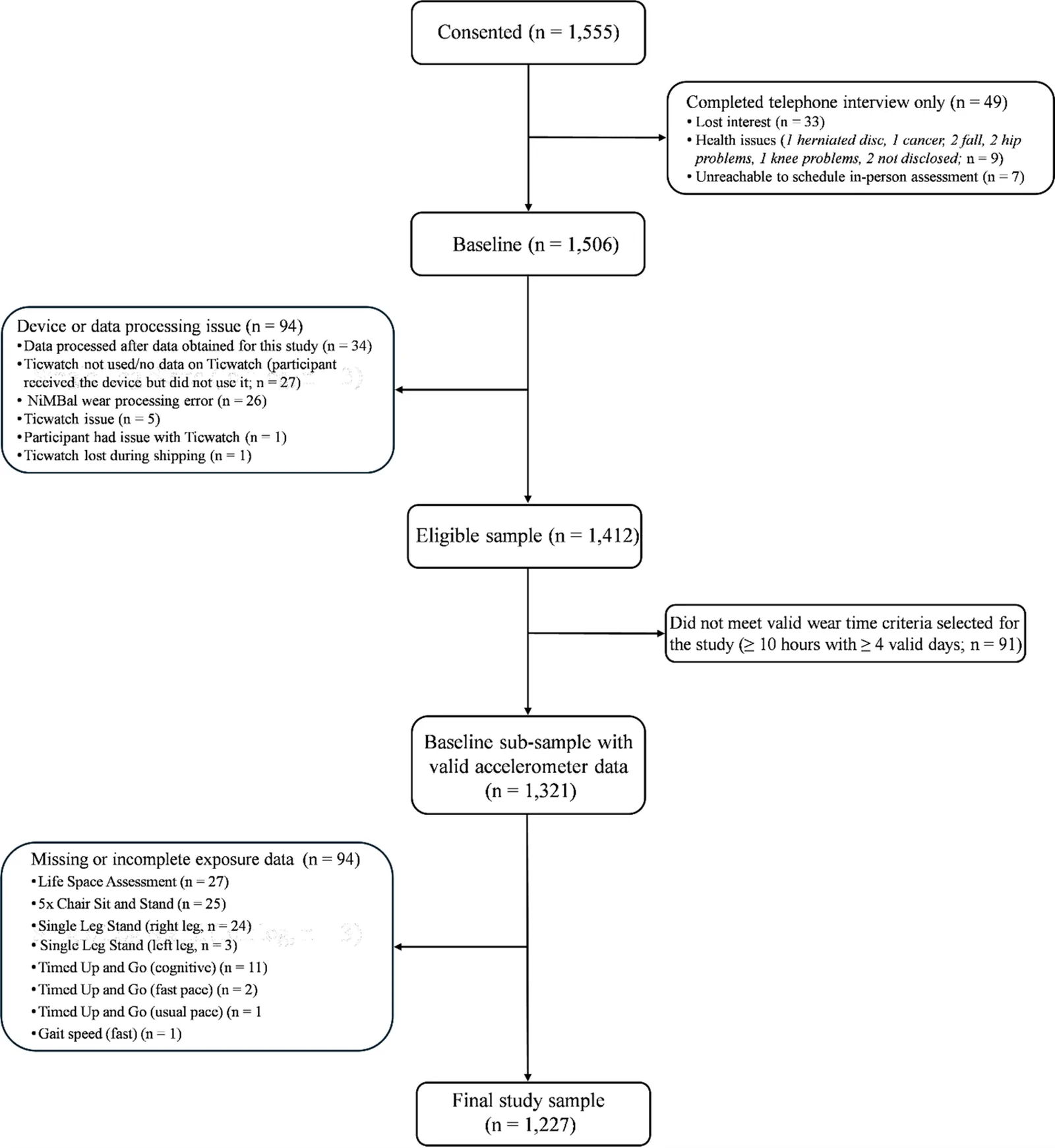
Flowchart of study participants

The final study sample included 1,227 participants (mean age = 73.6 years, SD = 5.3). A detailed flowchart summarising participant inclusion is presented in Fig. 1. Participants had an average of 9.5 (SD = 1.5) valid days and waking-day wear time ranged from 10.5 (minimum) to 18.9 (maximum) hours. Characteristics of the final study sample are in Table 2. A higher number of women (69.5%) were studied. Most participants had post-secondary education (76.9%), medium income level (41.8%) and normal cognitive function (83.1%). Confounder testing showed race, alcohol, smoking and marital status were not associated with any of the three behaviours; therefore, these variables were excluded in the final MANOVA models. The effect size for each mobility measure is provided in Supplementary Material Table A3.

**Table 2:** Participant characteristics

Characteristic	*N* or mean	% or SD
*Sex*		
Male	374	30.5
Female	852	69.5
*Age* (years)	73.6	5.3
*BMI*	26.2	4.7
*Education level*		
No post secondary education	284	23.1
Post-secondary degree/diploma	943	76.9
*Household income level*		
Low	376	30.6
Medium	513	41.8
High	338	27.5
*Chronic conditions (no.)*	3.3	1.9
*MoCA cognitive function score*		
Normal	1,020	83.1
Abnormal	207	16.9
*Use of walking aid*		
Yes	91	7.4
No	1,136	92.6

*BMI* body mass index, *MoCA* Montreal Cognitive Assessment, *SD* Standard deviation

The compositional means for SB, LIPA, and MVPA were 10:43, 2:11, 0:36, respectively (hours: minutes; Table 3). The highest co-dependence between pair-wise log ratios was LIPA and SB (0.74). The lowest co-dependence was between MVPA and SB (0.30) (Table 4).

**Table 3:** Descriptive characteristics of movement behaviours

Movement behaviour	Arithmetic mean (SD)	Median (IQR)	Compositional mean
SB	10:49 (1:29)	10:49 (1:57)	10:43
LIPA	2:21 (0:48)	2:20 (1:07)	2:11
MVPA	0:47 (0:31)	0:40 (0:41)	0:36
Total	13:57 (1:10)	13:58 (1:33)	13:30

Data presented in hours: minutes

**Table 4:** Variation matrix of correlations between movement behaviours

Movement behaviour	SB	LIPA	MVPA
SB	-	0.74	0.30
LIPA	0.74	-	0.73
MVPA	0.30	0.73	-

Descriptive characteristics of the mobility measures are in Table 5. Fifty-nine percent of participants (n = 731) were classified as having ‘no mobility limitation’ per the PCML. The median scores for the Physical Activity Scale for the Elderly (PASE) and LSA were 145.64 and 76, respectively. Median times were 8.85 s for the TUG, 12.28 s for the 5-Times Sit-to-Stand Test, and 4.63 min for the 400-meter walk test. Participants had a median gait speed of 1.26 m/second.

**Table 5:** Description of self-report and performance-based mobility measures

Mobility Measures	Median (IQR) or *n*
Self-report	
Preclinical Mobility Limitation Scale, score of 0,1, or 2	
* No mobility limitation (0)*	731
* Preclinical mobility limitation (1)*	200
* Minor manifest limitation (2)*	296
Late-Life Function and Disability Instrument-Disability Component, score 0-100 (↑score = ↓ disability)	54.49 (51.51–58.95)
Late-Life Function and Disability Instrument-Physical Function Component, score 0-100 (↑ score = ↑ function)	68.10 (62.51–74.59)
Physical Activity Scale for the Elderly, score 0-400 or more	145.64 (103.40-194.59)
Life Space Assessment, score 0-120	76 (64–90)
Mobility Assessment Tool-short form^†^, score 30–80	64.14 (57.82–64.14)
Performance-based	
Timed Up and Go (usual pace), seconds	8.85 (7.97–9.97)
Timed Up and Go (fast pace), seconds	6.31 (5.66–7.19)
Timed Up and Go (cognitive), seconds	9.22 (7.94–11.03)
Sit to Stand Test (5x), seconds	12.28 (10.53–14.37)
Gait speed (usual pace), meters/second	1.26 (1.10–1.39)
Gait speed (fast pace), meters/second	1.71 (1.55–1.87)
400-meter walk^†^, minutes	4.63 (4.23–5.14)
Single Leg Stance (right leg), seconds	17.38 (5.31–48.19)
Single Leg Stance (left leg), seconds	15.72 (5.12–45.47)
Stair-climbing power^†^, watts	247.58 (204.65-308.46)

^†^Tests performed only at Hamilton site. Sample sizes for the respective tests were *n* = 937; 899; and 919

Fifty-four associations reflecting three movement behaviours by 16 self-report and performance-based mobility measures and two indices were examined. Tables 6 and 7 present the back-transformed group means of time spent in each movement behaviour within each of the mobility measure groups, adjusted for age, sex, BMI, income level, education level, cognitive function, and number of chronic diseases. Table 8 presents a high-level summary of results for these associations to facilitate interpretation of overall patterns.

**Table 6:** Model-adjusted compositional means of movement behaviours by tertile of self-reported mobility measures

Self-report measures	SB	LIPA	MVPA
*Self-report measures index*			
Low	**10:13 (10:03**, 10:22)*	2:10 (2:07, 2:12)	**0:42 (0:39**,** 0:45)**
Moderate	9:49 (9:38, 10:00)	2:24 (2:22, 2:27)	0:57 (0:53, 1:02)
High	9:41 (9:31, 9:52)	2:29 (2:27, 2:31)	1:03 (0:59, 1:08)
*Preclinical Mobility Limitation Scale*			
No mobility limitation	**9:53 (9:46**, 9:59)*	2:22 (2:20, 2:23)	0:58 (0:55, 1:01)
Preclinical mobility limitation	9:54 (9:43, 10:05)	2:22 (2:19, 2:24)	0:53 (0:49, 0:57)
Minor limitation	10:12 (10:01, 10:22)	2:10 (2:08, 2:13)	**0:44 (0:41**, 0:48)*
*Late-Life Function and Disability Instrument (disability component)*			
Low	**10:07 (9:59**, 10:14)*	2:13 (2:12, 2:15)	**0:48 (0:45**, 0:51)*
Moderate	9:46 (9:37, 9:55)	2:27 (2:25, 2:29)	0:58 (0:55, 1:02)
High	9:57 (9:46, 10:07)	2:19 (2:17, 2:21)	0:56 (0:52, 1:00)
*Late-Life Function and Disability Instrument (function component)*			
Low	**10:05 (9:55**, 10:14)*	2:15 (2:13, 2:17)	**0:47 (0:43**, 0:50)*
Moderate	9:54 (9:44, 10:03)	2:22 (2:19, 2:23)	0:54 (0:51, 0:58)
High	9:52 (9:44, 10:00)	**2:21 (2:20**, 2:23)*	1:00 (0:56, 1:03)
*Physical Activity Scale for the Elderly*			
Low	**10:27 (10:18**, 10:34)*	1:59 (1:57, 2:01)	0:43 (0:41, 0:46)
Moderate	**9:59 (9:50**, 10:07)*	2:19 (2:17, 2:21)	0:49 (0:46, 0:52)
High	**9:41 (9:32**, 9:49)*	2:30 (2:28, 2:31)	**1:03 (0:59**, 1:06)*
*Life-Space Assessment*			
Low	10:02 (9:54, 10:10)	2:17 (2:15, 2:19)	0:49 (0:46, 0:52)
Moderate	10:02 (9:54, 10:10)	2:16 (2:14, 2:17)	0:53 (0:50, 0:56)
High	9:47 (9:31, 9:56)	2:25 (2:23, 2:27)	1:01 (0:58, 1:05)
*Mobility Assessment Tool-short form*			
Low	**10:11 (10:04**, 10:18)*	2:11 (2:09, 2:13)	**0:44 (0:42**, 0:47)*
Moderate	9:53 (9:43, 10:02)	2:22 (2:19, 2:25)	0:56 (0:51, 1:01)
High	9:49 (9:40, 9:59)	2:30 (2:27, 2:32)	1:00 (0:55, 1:05)

Data presented in hours:minutes(95%CI). Groups statistically significant from remaining groups are bolded and include an * symbol (*p* < 0.0167). Adjusted for age, sex, BMI, income level, education level, MoCA score, and number of chronic diseases. Interpretation example: Participants in the low tertile of the self-report measures index spent significantly more daily time in SB (10:13) compared to those in the moderate (9:49) and high (9:41) tertiles. ‘Low’, ‘moderate’, and ‘high’ refer to tertiles within each measure; interpretation differs depending on whether higher or lower values reflect better mobility performance

**Table 7:** Model-adjusted compositional means of movement behaviours by tertile of performance-based mobility measures

Performance-based measures	SB	LIPA	MVPA
*Performance-based measures index*			
Low	**9:36 (9:22**, 9:48)*	2:32 (2:30, 2:35)	**1:07 (1:02**, 1:13)*
Moderate	10:13 (10:03, 10:22)	2:10 (2:07, 2:12)	0:47 (0:41, 0:48)
High	10:04 (9:55, 10:09)	2:15 (2:13, 2:18)	0:49 (0:45, 0:52)
*Timed Up and Go (usual pace)*			
Low	**9:45 (9:36**, 9:54)*	2:26 (2:24, 2:27)	**1:03 (1:00**,** 1:07**)*
Moderate	10:03 (9:54, 10:12)	2:15 (2:13, 2:17)	0:51 (0:48, 0:55)
High	10:13 (10:04, 10:22)	2:09 (2:07, 2:11)	0:45 (0:42, 0:49)
*Timed Up and Go (fast pace)*			
Low	9:45 (9:35, 9:54)	2:26 (2:24, 2:27)	1:03 (0:59, 1:07)
Moderate	9:58 (9:48, 10:07)	2:19 (2:17, 2:20)	0:54 (0:51, 0:58)
High	**10:19 (10:11**, 10:27)*	2:06 (2:04, 2:07)	**0:41 (0:39**, 0:44)*
*Timed Up and Go (cognitive)*			
Low	**9:38 (9:27**, 9:49)*	2:29 (2:27, 2:31)	**1:09 (1:04**, 1:14)*
Moderate	10:02 (9:53, 10:11)	2:16 (2:14, 2:18)	0:52 (0:48, 0:55)
High	10:11 (10:04, 10:18)	2:11 (2:09, 2:12)	0:45 (0:43, 0:48)
*Sit to Stand Test (5x)*			
Low	9:44 (9:35, 9:53)	2:27 (2:25, 2:29)	1:02 (0:58, 1:06)
Moderate	9:57 (9:47, 10:06)	2:19 (2:17, 2:22)	0:54 (0:51, 0:58)
High	**10:14 (10:06**, 10:21)*	2:09 (2:07, 2:10)	**0:45 (0:42**, 0:47)*
*Gait Speed (usual pace)*			
Low	**10:18 (10:09**, 10:27)*	2:06 (2:03, 2:08)	**0:43 (0:40**, 0:46)*
Moderate	9:55 (9:37, 10:03)	2:21 (2:19, 2:22)	0:55 (0:52, 0:59)
High	9:40 (9:29, 9:51)	2:30 (2:28, 2:31)	1:04 (1:00, 1:09)
*Gait Speed (fast pace)*			
Low	10:13 (10:04, 10:21)	2:10 (2:08, 2:12)	**0:43 (0:40**, 0:46)*
Moderate	10:01 (9:51, 10:11)	2:16 (2:14, 2:18)	**0:54 (0:50**, 0:57)*
High	**9:37 (9:27**, 9:47)*	2:31 (2:29, 2:33)	**1:07 (1:03**, 1:11)*
*400-meter walk test*			
Low	**9:24 (9:11**, 9:36)*	2:39 (2:37, 2:41)	**1:14 (1:09**, 1:20)*
Moderate	**9:51 (9:40**, 10:01)*	2:24 (2:22, 2:26)	**0:54 (0:51**, 0:59)*
High	**10:22 (10:15**, 10:30)*	2:03 (2:00, 2:05)	**0:37 (0:35**, 0:40)*
*Single Leg Stance (right leg)*			
Low	10:09 (10:01, 10:17)	2:12 (2:10, 2:14)	**0:47 (0:44**, 0:50)*
Moderate	9:55 (9:46, 10:04)	2:21 (2:19, 2:22)	0:55 (0:52, 0:59)
High	9:48 (9:37, 9:58)	2:25 (2:23, 2:27)	0:58 (0:54, 1:02)
*Single Leg Stance (left leg)*			
Low	10:08 (10:01, 10:17)	2:12 (2:11, 2:14)	0:47 (0:42, 0:49)
Moderate	10:00 (9:51, 10:09)	2:17 (2:15, 2:19)	0:52 (0:49, 0:56)
High	9:53 (9:43, 10:03)	2:21 (2:19, 2:23)	0:58 (0:54, 1:02)
*Stair-climbing power*			
Low	**10:33 (10:21**, 10:44)*	1:56 (1:52, 1:59)	**0:38 (0:34**, 0:42)*
Moderate	10:01 (9:49, 10:11)	2:17 (2:14, 2:20)	0:53 (0:45, 0:57)
High	9:45 (9:32, 9:57)	2:28 (2:25, 2:31)	0:59 (0:54, 1:04)

Data presented in hours:minutes(95%CI). Groups statistically significant from remaining groups are bolded and include an * symbol (*p* < 0.0167). Adjusted for age, sex, BMI, income level, education level, MoCA score, and number of chronic diseases. Interpretation example: Participants in the ‘low’ tertile of Timed-Up and Go (usual pace) spent significantly less daily time in SB (9:45) compared to the moderate (10:03) and high (10:13) tertiles. ‘Low’, ‘moderate’, and ‘high’ refer to tertiles within each measure; interpretation differs depending on whether higher or lower values reflect better mobility performance

**Table 8:** Summary of results of the associations between mobility measures and movement behaviours relative to one another

Mobility measure	SB	LIPA	MVPA
Self-report measures index	↓	↔	↑
Preclinical Mobility Limitation Scale	↑	↔	↓
Late-Life Function and Disability Instrument- disability component	↓	↔	↑
Late-Life Function and Disability Instrument- function component	↓	↑	↑
Physical Activity Scale for the Elderly	↓	↔	↑
Life Space Assessment	↔	↔	↔
Mobility Assessment Tool-Short Form	↓	↔	↑
Performance-based measures index	↑	↔	↓
Timed Up and Go (usual pace)	↑	↔	↓
Timed Up and Go (fast pace)	↑	↔	↓
Timed Up and Go (cognitive)	↑	↔	↓
Repeated Chair-Stand	↑	↔	↓
Gait speed (usual pace)	↓	↔	↑
Gait speed (fast pace)	↓	↔	↑
400-meter walk test	↑	↔	↓
Single-Leg Stance, right leg	↔	↔	↑
Single-Leg Stance, left leg	↔	↔	↔
Stair-Climbing Power	↓	↔	↑

↑ indicates a significant positive association, ↓ indicates a significant negative association, ↔ indicates a non-significant association (*p* < 0.0167 for all)

There were significant differences in waking-day movement behaviour composition across tertiles of the self-report and performance-based mobility measures and indices. Older adults with lower mobility consistently demonstrated more SB and less MVPA per day across all self-report measures except LSA. For instance, those in the low PASE tertile accumulated an additional 46 min/day of SB compared to the high tertile (*p* < 0.001). Conversely, those in the high PASE tertile accumulated 20 more minutes/day of MVPA than those in the low tertile (*p* < 0.001). The best performing self-report measure for explaining waking-day movement behaviour composition was the PASE (*partial η*^2^ = 0.03, *p* < 0.001).

For all performance-based measures except the single leg stance, older adults with better mobility consistently demonstrated lower daily SB and higher MVPA. The three TUG tests showed that those in the low tertile (i.e., faster completion times, indicating better performance) spent less time in SB per day compared to the high tertile. For instance, the low TUG usual pace tertile had 28 fewer minutes/day of SB than the high tertile (*p* < 0.001). Conversely, that same tertile accumulated 18 more minutes/day of MVPA compared to the high tertile (*p* < 0.001). The 400-meter walk test was the most consistent performance-based measure showing significant differences across all tertiles for both SB and MVPA. For SB, participants in the low tertile (fastest completion times) spent 27 and 58 fewer minutes/day than the moderate (*p* < 0.001) and high (*p* < 0.001) tertiles, respectively. For MVPA, those in the low tertile accumulated 20 and 37 more minutes/day than the moderate (*p* < 0.001) and high (*p* < 0.001) tertiles, respectively. The 400-meter walk test was also the best performance-based measure for capturing waking-day movement behaviour composition (*partial η²* = 0.07, *p* < 0.001).

The LLFDI function component was the only measure to show significant group differences in LIPA. Older adults in the high tertile accumulated six more minutes/day of LIPA compared to the low tertile (*p* = 0.017).

Both self-report and performance-based measure indices demonstrated significant group differences in daily SB and MVPA. However, the self-reported measures index showed greater differences in both behaviours. For SB, the low tertile of the self-reported index had 32 fewer minutes/day than the high tertile (*p <* 0.001) compared to 28 fewer minutes/day for the performance-based measures index (*p* < 0.001). For MVPA, the low tertile of the self-reported measures index had 21 fewer minutes/day than the high tertile (*p* < 0.001), while it was 18 fewer minutes/day for the performance-based measures index (*p* < 0.001). Both indices had the same effect size for capturing waking-day movement behaviour composition (*partial η²* = 0.02, *p* < 0.001).

## Discussion

To our knowledge, this is the first study to use CoDA to examine associations between commonly used self-report and performance-based mobility measures, and the waking-day movement behaviour composition in older adults. Differences in waking-day movement behaviour composition were found across the self-report and performance-based mobility measures. Older adults with lower mobility consistently accumulated more SB and less MVPA throughout the day. However, the association with different movement behaviours varied depending on the specific measure, with the PASE and 400-meter walk test identified as the best self-report and performance-based measures. Overall, the observed associations between the mobility measures and waking-day movement behaviours support the validity of commonly used mobility measures in identifying meaningful differences in daily movement behaviour composition; however, the small effect sizes indicate limited ability to explain or predict real-world mobility. Differences ranged from small (e.g., 1 min/day of LIPA or about 0.01% difference) to large (58 min/day of SB or about 7% difference), followed a graded pattern across tertiles and were directionally consistent (e.g., SB was highest in the lowest gait speed tertile).

While it is difficult to directly compare findings from studies using conventional statistics with those derived from CoDA, ours showed that both self-report and performance-based measures of mobility are related to device-derived waking-day movement behaviour composition. Among the performance-based measures, the most demanding tests of physical capacity, namely the 400-meter walk, and fast gait speed were the most sensitive indicators of how older adults spend their time in waking-day movement behaviours. Similar results on the relationship between these two tests and accelerometer derived daily physical activity have been found in other studies. For instance, we observed a strong and consistent negative association between 400-meter walk times and accumulated daily MVPA across low, moderate, and high tertiles. A study examining a sample of German older adults (mean age = 80.8 years) found that faster 400-meter walk time is associated with higher daily step count (*β* = -6.4, 95%CI: -9.99, -3.3) and cadence (*β* = -0.018, 95%CI: -0.028, -0.009) [63]. Further, we observed a similar relationship between fast gait speed and daily MVPA across all tertiles in our sample. A study assessing older adults with a mean age of 73 years found a similar negative association between fast gait speed and daily MVPA in women (β = 0.24, 95%CI: 0.18, 0.30) and men (β = 0.18, 95%CI: 0.07, 0.20) [28]. Our results suggest faster walk completion time and higher gait speed are associated with meaningful increase in daily MVPA. Furthermore, the effect sizes of these two tests, although modest, were the largest estimates among those we tested, suggesting they may be more informative over the other measures. One possible explanation is that these tests involve sustained and purposeful movement, an essential component of daily activity in older adults, as opposed to brief or static assessments that take place in a controlled setting (e.g., single leg balance). Moreover, they integrate multiple physiological systems, namely anaerobic and aerobic capacity, lower limb strength, dynamic balance and stability, all of which are important for real-world mobility. These stronger associations may therefore reflect construct alignment with device-detected ambulatory activity, rather than true superiority as indicators of overall mobility [64].

We observed numerous significant associations between mobility level and waking-day movement behaviours, particularly among individuals in the lowest performing tertiles and their engagement in daily SB with 10 of the 18 mobility measures showing significant associations. Notably, the PASE and 400-meter walk test were the strongest measures of daily SB and demonstrated the greatest magnitude in differences whereby individuals in the lowest performing tertile spent on average, 45 more minutes per day in SB compared to the highest tertile. Our finding that PASE strongly explains daily SB differs from previous studies [65–67]. For instance, a study assessing American older adults (median age = 71 years) found no significant relationship between PASE score and accelerometry derived total SB minutes per day (*r* = 0.04, *p* = 0.699) [67]. The strong negative association we observed between PASE score and daily SB suggest that PASE may have greater utility in capturing differences in SB when analysed using CoDA, because it accounts for the important co-dependence between movement behaviours that is not considered with traditional statistical methods.

A noteworthy observation of this study is that none of the mobility measures showed a significant association with LIPA, with the one exception being a marginal association observed in the highest tertile on the LLFDI-function component. The current literature on the relationship between mobility performance and daily time spent in LIPA is conflicting [25, 26, 68–70]. Using a CoDA framework, our study is the first to demonstrate no meaningful relationship between either self-report and performance-based measures of mobility and daily LIPA. The lack of observed association may reflect a systematic measurement issue in existing tools, that may not be sensitive enough to detect light intensity and incidental physical activity. The increasing credence given to LIPA in movement behaviour guidelines and public health messaging aimed at older adults [22, 71, 72], many of whom may be unwilling or unable to perform MVPA highlights there is a need to evaluate mobility with more ecologically valid tools. For instance, going beyond conventionally accepted minimum wear times or wearing both wrist and thigh accelerometers simultaneously may be necessary to enhance estimates of older adults’ real-world mobility.

Our results must be interpreted in light of the limitations. First, the cross-sectional design precludes making causal inferences. Further, the findings may be most generalisable to higher functioning older adults as the median values for TUG, gait speed and 400-meter walk test indicated above-average physical performance [44, 73, 74] consistent with a sample of older adults without major mobility limitations at baseline [37]. Also, the study addressed waking-day movement behaviour composition, as data on sleep was not collected. This approach is consistent with other CoDA studies that did not have sleep data [75]. Further studies that include the full 24-h movement profile are needed to validate our findings. Last, although accelerometers provide direct measures of movement behaviours, wrist-worn devices cannot accurately capture posture (e.g., sitting vs. standing), which may lead to some misclassification between SB and LIPA and attenuate effect estimates [76]. While we have shown that certain self-report and performance-based mobility measures, specifically PASE, 400-meter walk test, and fast gait speed, perform well for capturing the waking-day movement behaviour composition in relation to MVPA and SB, the next step is to extend these observations to a more diverse sample using longitudinal study design that also considers device-based measures of mobility.

## Conclusion

In summary, traditional self-report and performance-based mobility measures were associated with the waking-day movement behaviour composition in older adults; however, the small effect sizes indicate a limited ability to fully capture real-world behaviour. There was a graded pattern across mobility level and daily time spent in SB and MVPA, respectively. The PASE, 400-meter walk and fast gait speed tests offered the clearest indication of how older adults distribute their time across waking-day movement behaviours. Overall, our findings raise important questions about the applicability of traditional mobility measures for making inferences about real-world mobility. None of the 18 tested measures captured daily LIPA despite being the activity intensity older adults spend the most of their time in. Future studies should use CoDA to evaluate other potential mobility measures, including device-based metrics, and their relationship with the daily movement behaviour composition in older adults.

## Supplementary Information

Additional file 1: Table A1. Boundary values between the low, moderate and high tertiles for each mobility measure. Table A2. Self-reported chronic conditions diagnosed by a doctor for chronic conditions variable. Table A3. Partial η² effect sizes of self-report and performance-based mobility measures in capturing waking-day movement behaviour composition. Figure A1. Results of power analysis.

Additional file 2: STROBE checklist.

## Data Availability

The datasets used and/or analysed during the current study are available from the corresponding author on reasonable request.

## Abbreviations

AVM: Average vector magnitude
BMI: Body mass index
CI: Confidence interval
CoDA: Compositional data analysis
DETACH: Device Temperature and Accelerometer Change
ICF: International Classification of Functioning, Disability and Health
ilr: Isometric log ratio
IQR: Interquartile range
LIPA: Light-intensity physical activity
LLFDI: Late-Life Function and Disability Instrument
LSA: Life Space Assessment
MacM3: McMaster Monitoring my Mobility study
MANOVA: Multivariate analysis of variance
MoCA: Montreal Cognitive Assessment
MVPA: Moderate-to-vigorous physical activity
PASE: Physical Activity Scale for the Elderly
PCML: Preclinical Mobility Limitation
SB: Sedentary behaviour
SD: Standard deviation
TUG: Timed Up and Go
