# Supplementary material for: Mobility measures and waking-day movement behaviour composition among older adults: a compositional data analysis of the McMaster Monitoring My Mobility study
Source: Int J Behav Nutr Phys Act. 2026 Jun 23;23:84. doi: 10.1186/s12966-026-01939-4 (PMC13548552; doi:10.1186/s12966-026-01939-4)
Supplement: Supplementary file 1 [file 12966_2026_1939_MOESM1_ESM.docx]

Table A1. Boundary values between the low, moderate and high tertiles for each mobility measure*.*

| **Mobility measure** | **Tertile** | | |
| --- | --- | --- | --- |
|  | **Low** | **Moderate** | **High** |
| Self-report measures index | ≤ 41.95 | > 41.95 - ≤ 60.93 | > 60.93 |
| Preclinical Mobility Disability Scale | Predefined category | Predefined category | Predefined category |
| Late-Life Function and Disability Instrument-  disability component, score 0-100 | ≤ 52.23 | > 52.23 - ≤ 57.04 | > 57.04 |
| Late-Life Function and Disability Instrument-  function component, score 0-100 | ≤ 64.48 | > 64.48 - ≤ 72.31 | > 72.31 |
| Physical Activity Scale for the Elderly, score 0-400 or more | ≤ 116.95 | > 116.95 - ≤ 176.81 | > 176.81 |
| Life Space Assessment, score 0-120 | ≤ 69.24 | > 69.24 - ≤ 84 | > 84 |
| Mobility Assessment Tool-Short Form, score 30-80 | ≤ 61.01 | > 61.01 - ≤ 64.14 | > 64.14 |
| Performance-based measures index | ≤ 45.73 | > 45.73 - ≤ 52.53 | > 52.53 |
| Timed Up and Go (usual pace), seconds | ≤ 8.22 | > 8.22 - ≤ 9.56 | > 9.56 |
| Timed Up and Go (fast pace), seconds | ≤ 5.90 | > 5.90 - ≤ 6.90 | > 6.90 |
| Timed Up and Go (cognitive), seconds | ≤ 8.35 | > 8.35 - ≤ 10.32 | > 10.32 |
| Repeated Chair-Stand, seconds | ≤ 11.09 | > 11.09 - ≤ 13.53 | > 13.53 |
| Gait speed (usual pace), meters/second | ≤ 1.14 | > 1.14 - ≤ 1.35 | > 1.35 |
| Gait speed (fast pace), meters/second | ≤ 1.62 | > 1.62 - ≤ 1.80 | > 1.80 |
| 400-meter walk test, minutes | ≤ 4.37 | > 4.37 - ≤ 4.97 | > 4.97 |
| Single-Leg Stance (right leg), seconds | ≤ 8.20 | > 8.20 - ≤ 33.96 | > 33.96 |
| Single-Leg Stance (left leg), seconds | ≤ 7.45 | > 7.45 - ≤ 30.70 | > 30.70 |
| Stair-Climbing Power, watts | ≤ 221.84 | > 221.84 - ≤ 286.70 | > 286.70 |

Table A2. Self-reported chronic conditions diagnosed by a doctor for chronic conditions variable.

| Chronic_1: Vision | 1. Macular degeneration  2. Cataracts  3. Glaucoma |
| --- | --- |
| Chronic_2: Gastrointestinal | 4. Bowel Incontinence  5. Urinary Incontinence |
| Chronic_3: Musculoskeletal | 6. Osteoarthritis  7. Osteoporosis  8. Back pain  9. Chronic pain |
| Chronic_4: Neurological | 10. Memory problem  11. Dementia or Alzheimer’s  12. Multiple sclerosis  13. Epilepsy  14. Stroke/Cerebrovascular accident  15. Ministroke/Transient ischemic attack  16. Traumatic brain injury  17. Parkinsonism |
| Chronic_5: Cardiac/cardiovascular | 18. Heart disease (incl. congestive heart failure)  19. Peripheral arterial disease  20. Hypertension/high blood pressure  21. Angina  22. Heart attack/myocardial infarction  23. Aortic valve stenosis |
| Chronic_6: Mental health | 24. Anxiety disorder  25. Mood disorder  26. Clinical depression  27. Depression |
| Chronic_7: Respiratory | 28. Asthma  29. Chronic obstructive pulmonary disorder  30. Bronchitis  31. Emphysema |
| Chronic_8: Other conditions | 32. Kidney disease/failure  33. Diabetes  34. Cancer |

Table A3. Partial η² effect sizes of self-report and performance-based mobility measures in capturing waking-day movement behaviour composition.

| Mobility Measure | Effect size  (*partial η*^2^) | p-value |
| --- | --- | --- |
| Self-report measures index | 0.02 | < 0.001 |
| Preclinical Mobility Disability Scale | 0.01 | 0.007 |
| Late-Life Function and Disability Instrument-  disability component | 0.01 | 0.004 |
| Late-Life Function and Disability Instrument-  function component | 0.01 | 0.008 |
| Physical Activity Scale for the Elderly | 0.03 | < 0.001 |
| Life Space Assessment | 0.01 | 0.003 |
| Mobility Assessment Tool-Short Form | 0.02 | 0.005 |
| Performance-based measures index | 0.02 | 0.001 |
| Timed Up and Go (usual pace) | 0.02 | 0.004 |
| Timed Up and Go (fast pace) | 0.03 | < 0.001 |
| Timed Up and Go (cognitive) | 0.03 | < 0.001 |
| Repeated Chair-Stand | 0.02 | < 0.001 |
| Gait speed (usual pace) | 0.02 | < 0.001 |
| Gait speed (fast pace) | 0.03 | < 0.001 |
| 400-meter walk test | 0.07 | < 0.001 |
| Single-Leg Stance, right leg | 0.01 | 0.05 |
| Single-Leg Stance, left leg | 0.01 | 0.186 |
| Stair-Climbing Power | 0.03 | < 0.001 |

Figure A1. Results of power analysis.
