# Supplementary material for: Mobility measures and waking-day movement behaviour composition among older adults: a compositional data analysis of the McMaster Monitoring My Mobility study
Source: Int J Behav Nutr Phys Act. 2026 Jun 23;23:84. doi: 10.1186/s12966-026-01939-4 (PMC13548552; doi:10.1186/s12966-026-01939-4)
Supplement: Supplementary file 2 [file 12966_2026_1939_MOESM2_ESM.docx]

STROBE Statement—checklist of items that should be included in reports of observational studies

|  | Item No. | Recommendation | Page  No. | | Relevant text from manuscript |
| --- | --- | --- | --- | --- | --- |
| **Title and abstract** | 1 | (*a*) Indicate the study’s design with a commonly used term in the title or the abstract | Abstract, p.2 | | - A cross-sectional analysis of data from over 1200 older adults… |
|  |  | (*b*) Provide in the abstract an informative and balanced summary of what was done and what was found | Abstract, p.2 | | - Compositional data analysis was used…(methods) - Significant differences in movement behaviour composition were found…(results) - Older adults with lower mobility…(results) - Traditional self-report and performance-based measures of mobility are associated with…(conclusions) |
| Introduction | | | | |  |
| Background/rationale | 2 | Explain the scientific background and rationale for the investigation being reported | Background, p.4-6 | | - Mobility is a cornerstone of healthy ageing… - Practitioners use a range of self-report and performance-based measures… - Their validity for providing insights into real-world, everyday mobility remain unclear… |
| Objectives | 3 | State specific objectives, including any prespecified hypotheses | Background, p.6 | | - The objectives of this study are to use a CoDA framework to… |
| Methods | | | | |  |
| Study design | 4 | Present key elements of study design early in the paper | Methods: S*tudy design and participants*, p.6 | | - This study is a cross-sectional analysis… |
| Setting | 5 | Describe the setting, locations, and relevant dates, including periods of recruitment, exposure, follow-up, and data collection | Methods: S*tudy design and participants*, p.6; *Mobility measures*, p.7 | | - The study includes 1,506 older adults from Southern Ontario, Canada. - Baseline data collected from 2022… - Mobility measures collected over the phone or in person… |
| Participants | 6 | (*a*) *Cohort study*—Give the eligibility criteria, and the sources and methods of selection of participants. Describe methods of follow-up  *Case-control study*—Give the eligibility criteria, and the sources and methods of case ascertainment and control selection. Give the rationale for the choice of cases and controls  *Cross-sectional study*—Give the eligibility criteria, and the sources and methods of selection of participants | Methods: S*tudy design and participants*, p.6 | | - Inclusion criteria were… |
|  |  | (*b*) *Cohort study*—For matched studies, give matching criteria and number of exposed and unexposed  *Case-control study*—For matched studies, give matching criteria and the number of controls per case |  | | N/A |
| Variables | 7 | Clearly define all outcomes, exposures, predictors, potential confounders, and effect modifiers. Give diagnostic criteria, if applicable | Methods: *Mobility measures*, p.6-7; *Movement behaviour variables*, p*.*7-8*; Covariates*, p.8 | | - The explanatory variables are… - The outcome variables were… - Covariates were considered… |
| Data sources/ measurement | 8* | For each variable of interest, give sources of data and details of methods of assessment (measurement). Describe comparability of assessment methods if there is more than one group | Methods: *Mobility measures*, p.6-7; *Movement behaviour variables*, p.7-8; *Covariates*, p. 9; *Table 1*, p.12 | | - The explanatory variables are measures of mobility… - The outcome variables were the relative proportions of the waking day… - Covariates were considered… - Table 1 summarises the mobility measures, briefly describes them and lists the data source. |
| Bias | 9 | Describe any efforts to address potential sources of bias | Methods: *Movement behaviour variables*, p.7  References: #34 and 36, p.33 | | ***Rationale:***   - Raw accelerometer data was processed using the NiMBalWear analytic pipeline which may have reduced measurement bias. - This study uses baseline data from the MacM3 study. A multipronged recruitment strategy was used aimed at enhancing the diversity of participants in the study (ethnicity, race, income level, culture, ability and region) to enhance generalisability of the cohort and reduce selection bias.   The MacM3 study and NiMBalWear algorhithm are fully described elsewhere and cited in the article, respectively. |
| Study size | 10 | Explain how the study size was arrived at | Methods: *Figure 1-* Participant flowchart, p.6; *Analysis strategy*, p.9; Appendix, Figure A1. | | - Figure 1 depicts how the final study sample was arrived at and provides exclusion reasons. - *A posteriori* power analysis was conducted… |
| Quantitative variables | 11 | Explain how quantitative variables were handled in the analyses. If applicable, describe which groupings were chosen and why | Methods: *Mobility measures*, p.6-7; *Analysis strategy,* p. 9; *Covariates*, p.8; *Analysis strategy*, p.10-11 | - …‘low’, ‘moderate’ and ‘high’ levels of mobility, scores for each mobility measure were grouped into tertiles. - …isometric log ratios were used to transform movement behaviour variables from their natural space to constrained space so that they can be appropriately analysed with standard techniques. - Multiple covariates were incorporated, preserved in their continuous form when appropriate, with alternative categorical or transformed specifications evaluated only when warranted (e.g., income level) - Sex and age stratified analyses were run (male/female; mean age split < 73.6 years ≥ 73.6 years) based on their *a priori* consideration as effect modifiers. | |
| Statistical methods | 12 | (*a*) Describe all statistical methods, including those used to control for confounding | Methods: *Analysis strategy*, p*.* 9-10 | - Compositional data analysis was used to determine differences in waking day movement behaviour composition… - … the two *ilr* coordinates of each movement behaviour were entered as the outcome variables in multivariate analysis of variance (MANOVA) models run for each mobility measure. - …the α level was set to 0.0167 using the Bonferroni correction. - Confounding variables were tested against each movement behaviour using univariate linear regression. Those significantly associated with any behaviour based on a conservative p-value of < 0.2 were controlled for… | |
|  |  | (*b*) Describe any methods used to examine subgroups and interactions | Methods: *Analysis strategy*, p.10-11 | - Sex and age stratified analyses were run based on their *a priori* consideration as effect modifiers. | |
|  |  | (*c*) Explain how missing data were addressed | Methods: p.6: *Figure 1: flowchart of study participants* | - 94 participants (6% of sample) with missing or incomplete exposure data were excluded from the final analysis. Details are provided in the flowchart. | |
|  |  | (*d*) *Cohort study*—If applicable, explain how loss to follow-up was addressed  *Case-control study*—If applicable, explain how matching of cases and controls was addressed  *Cross-sectional study*—If applicable, describe analytical methods taking account of sampling strategy | Methods: *Study design and participants*, p.6 | N/A-This study is s cross-sectional analysis of baseline data from The MacM3 study which was gathered using convenience sampling. Reference to the cohort profile is provided in the Methods (ref #34). | |
|  |  | (*e*) Describe any sensitivity analyses |  | N/A | |
| Results | | | | | |
| Participants | 13* | (a) Report numbers of individuals at each stage of study—e.g. numbers potentially eligible, examined for eligibility, confirmed eligible, included in the study, completing follow-up, and analysed | Methods: *Study design and participants*, Figure 1, p.6 | Please see flow diagram for specific exclusion reasons. | |
|  |  | (b) Give reasons for non-participation at each stage |  |  | |
|  |  | (c) Consider use of a flow diagram | Methods: Figure 1, p.6 | - A flow diagram detailing participant inclusion is provided. | |
| Descriptive data | 14* | (a) Give characteristics of study participants (eg demographic, clinical, social) and information on exposures and potential confounders | Results: p.13; Table 2, p.16; Table 3, p.16; Table 4, p.16, Table 5, p.17 | - Characteristics of the final study sample are in Table 2… a higher number of women (69.5%) were studied… - The compositional means for SB, LIPA, and MVPA were…(Table 3) - The highest co-dependence between pair-wise log ratios were…(Table 4) - Descriptive characteristics of the mobility measures are in Table 5… | |
|  |  | (b) Indicate number of participants with missing data for each variable of interest | Methods, Figure 1: *Flowchart of study participants*, p.6 | - There were no participants with missing outcome data. There were 94 participants (6% of the sample) with missing exposure data who were excluded from the final analysis. The flowchart highlights the number of participants missing for each exposure variable. | |
|  |  | (c) *Cohort study*—Summarise follow-up time (e.g., average and total amount) |  | N/A | |
| Outcome data | 15* | *Cohort study*—Report numbers of outcome events or summary measures over time |  |  | |
|  |  | *Case-control study—*Report numbers in each exposure category, or summary measures of exposure |  |  | |
|  |  | *Cross-sectional study—*Report numbers of outcome events or summary measures | Results; Table 5, p.10; Tables 6 and 7, p.18-19; Table 8, p. 20 | - Descriptive characteristics of the mobility measures are in Table 5… - Tables 6 and 7 present the back-transformed group means of time spent in each movement behaviour… - Table 8 presents a summary of results for these associations. | |
| Main results | 16 | (*a*) Give unadjusted estimates and, if applicable, confounder-adjusted estimates and their precision (eg, 95% confidence interval). Make clear which confounders were adjusted for and why they were included | Results; p.13-14; Tables 6 and 7, p. 18-19  Methods: *Covariates*, p. 17; *Analysis strategy*, p.10; Results, p.13 | - Tables 6 and 7 present the back-transformed group means…adjusted for age, sex, BMI, income level, education level, cognitive function, and number of chronic diseases. - Confounder testing showed race, alcohol, smoking and marital status were not associated with any of the three behaviours… - Covariates were considered based on their known relationship… - Confounding variables were tested against each movement behaviour using univariate linear regression. Those significantly associated…based on a conservative p-value of < 0.2 were controlled for… | |
|  |  | (*b*) Report category boundaries when continuous variables were categorized | Results, Table 5, p. 17 | - Descriptive characteristics of the mobility measures are in Table 5. | |
|  |  | (*c*) If relevant, consider translating estimates of relative risk into absolute risk for a meaningful time period |  | N/A | |
| Other analyses | 17 | Report other analyses done—eg analyses of subgroups and interactions, and sensitivity analyses | Methods: *Analysis strategy*, p.10-11 | - Sex and age stratified analyses were run (male/female; mean age split < 73.6 years ≥ 73.6 years)… - The patterns of association were generally the same as the full sample…final analyses were run on the full sample with sex and age treated as confounding variables. | |
| Discussion | | | | | |
| Key results | 18 | Summarise key results with reference to study objectives | Discussion, p.21 | - Differences in waking day movement behaviour composition were found… - Older adults with lower mobility consistently accumulated more SB and less MVPA… - … the PASE being the best self-report measure and the 400-meter walk test the best performance-based measure. - …the mobility measures were found to be weak measures of real-world mobility. | |
| Limitations | 19 | Discuss limitations of the study, taking into account sources of potential bias or imprecision. Discuss both direction and magnitude of any potential bias | Discussion, p.23-24 | - the cross-sectional design precludes making causal inferences… - … the generalisability of the findings on the applicability of self-report and performance-based measures… - the study addressed waking day movement behaviour composition, as data on sleep was not collected… - wrist-worn devices cannot accurately capture posture (e.g., sitting vs. standing), which may lead to some misclassification… | |
| Interpretation | 20 | Give a cautious overall interpretation of results considering objectives, limitations, multiplicity of analyses, results from similar studies, and other relevant evidence | Discussion, p.21-24 | - …this is the first study to… - The significant differences that existed ranged in magnitude from small to large…followed a dose-response gradient and… were in the expected direction. - These results support the validity of commonly used mobility measures… - …similar results…have been found in other studies. - Our finding that PASE strongly predicts daily SB differs from previous studies. - While we have shown that…the next steps are to… - Future studies should… | |
| Generalisability | 21 | Discuss the generalisability (external validity) of the study results | Discussion, p. 23 | - … the generalisability of the findings… | |
| Other information |  | | | | |
| Funding | 22 | Give the source of funding and the role of the funders for the present study and, if applicable, for the original study on which the present article is based | Declarations: *Funding*, p.26 | - S.H. is funded by… - The funders had no role… | |

*Give information separately for cases and controls in case-control studies and, if applicable, for exposed and unexposed groups in cohort and cross-sectional studies.

**Note:** An Explanation and Elaboration article discusses each checklist item and gives methodological background and published examples of transparent reporting. The STROBE checklist is best used in conjunction with this article (freely available on the Web sites of PLoS Medicine at http://www.plosmedicine.org/, Annals of Internal Medicine at http://www.annals.org/, and Epidemiology at http://www.epidem.com/). Information on the STROBE Initiative is available at www.strobe-statement.org.
